# Supplementary material for: The interactome of intact mitochondria by cross-linking mass spectrometry provides evidence for coexisting respiratory supercomplexes
Source: Mol Cell Proteomics. 2017 Dec 8;17(2):216–32. doi: 10.1074/mcp.RA117.000470 (PMC5795388; doi:10.1074/mcp.RA117.000470)
Supplement: Supplemental Data [file supp_RA117.000470_133922_0_supp_23978_fzffwf.zip › spectra_annotation/mito_DR_spectra_annotation/401-1-10-1-11-1.pdf]

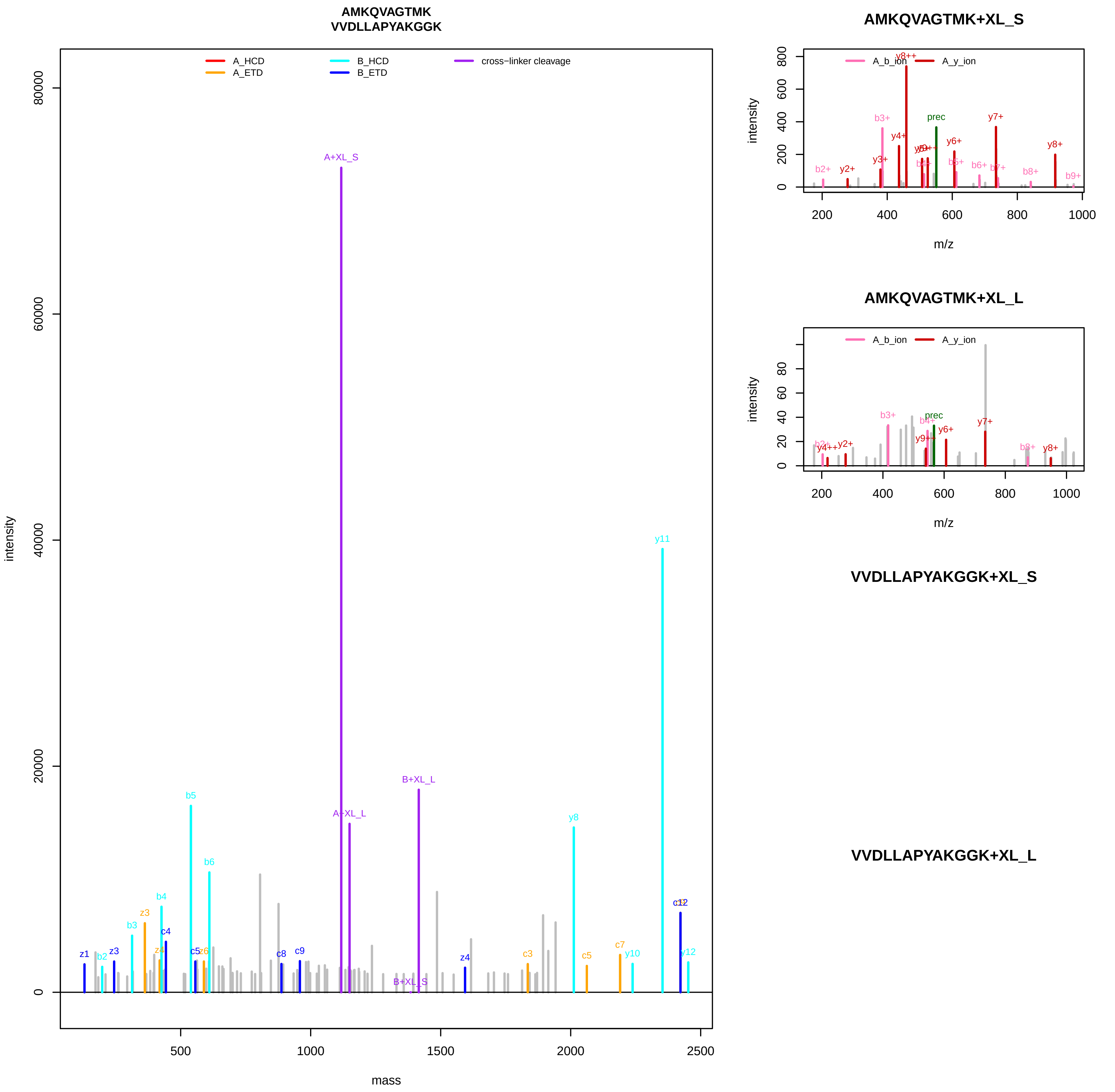

AMKQVAGTMK
VVDLLAPYAKGGK
A_HCD
A_ETD
B_HCD
B_ETD
cross−linker cleavage
A+XL_S
A+XL_L
B+XL_L
B+XL_S
z1
b2
z3
b3
z3
b4
c4
z4
b5
c5
z6
b6
c8
c9
z4
c3
y8
c5
c7
y10
y11
c12
y12
intensity
mass
0
20000
40000
60000
80000
500
1000
1500
2000
2500
AMKQVAGTMK+XL_S
A_b_ion
A_y_ion
b2+
y2+
y3+
b3+
y4+
y8++
prec
y6+
y7+
y8+
b6+
b8+
b9+
intensity
m/z
AMKQVAGTMK+XL_L
A_b_ion
A_y_ion
b3+
prec
y6+
y7+
y2+
b8+
y8+
intensity
m/z
VVDLLAPYAKGGK+XL_S
VVDLLAPYAKGGK+XL_L
